# The Spanish Political Attitudes Panel (12 waves)

**DOI:** 10.1038/s41597-025-05684-4

**Published:** 2025-09-26

**Authors:** Roberto Pannico, Carolina Galais, Enrique Hernández, Guillem Rico, Eva Anduiza

**Affiliations:** Universitat Autònoma de Barcelona, Barcelona, Spain

**Keywords:** Politics, Society

## Abstract

The online POLAT Panel (Spanish Political Attitudes Dataset) tracks changes in the political attitudes and behaviors of 4,633 Spanish individuals over a 10-year period. Unlike most panel surveys that primarily emphasize socio-economic characteristics, the POLAT Panel focuses mainly on political variables. Conducted across 12 waves from 2010 to 2020, the dataset provides valuable longitudinal insights into how citizens’ political views change over time. It allows researchers to analyze shifts in public opinion in the context of significant events such as the financial crisis and the associated wave of protests, the rise of populism and the appearance of new political parties, or the COVID-19 pandemic. By tracking individual-level changes over time, the POLAT Panel provides a rich resource for understanding how political attitudes are shaped by both individual experiences and societal events.

## Background & Summary

The POLAT Panel was designed to track changes in individuals’ political attitudes and behavior over time. This longitudinal approach helps researchers understand how personal experiences, aging, changing social contexts or political events influence individuals’ political opinions, political engagement and voting behavior. Panel data are important in revealing underlying trends and evolving patterns that might be missed in cross-sectional studies. By observing the same individuals at multiple points in time, panel data can be used to explore how political attitudes are formed, evolve, change or stabilize. They also provide more leverage to test causal relations, as focusing on within-individual variation can rule out any confounders that are time-invariant within individuals.

Many long-standing panel surveys, such as the American Panel Study of Income Dynamics (PSID) or the British Household Panel Survey (BHPS), have focused predominantly on socio-economic and demographic factors. These panels only offer a limited set of political variables, restricted to a small number of basic indicators such as party identification, left-right placement, or political interest. The POLAT Panel helps fill this gap by providing extensive longitudinal data on a wide range of political attitudes and behaviors. In terms of question coverage, it is comparable to other panel surveys such as the Dutch Longitudinal Internet studies for the Social Sciences (LISS)^1^, the Norwegian Citizen Panel (NCP)^2^, or two British panels: the NatCen Panel^3^ and BES Internet Panel^4^. Some other panels with political content exist, but are shorter in length, e.g. the TRI-POL project^5^ or the Israel Polarization Panel^6^. The POLAT Panel offers a unique perspective by providing a decade’s worth of longitudinal data from a Southern European country that has experienced intense social and economic upheaval. Moreover, it features an adaptive design, with a set of recurring questions and various modules that are activated according to the political circumstances of the moment.

The POLAT Panel’s extended time span and its focus on Spain provides a unique opportunity to track and analyze shifts in citizens’ political attitudes and behaviors in relation to both global and local events. Key global events that took place during the ten years of the panel include the financial crisis and subsequent European debt crisis, the wave of protests generated by austerity measures, the rise of populist, radical right and radical left parties, or the COVID-19 pandemic. Events that are more closely tied to the Spanish case include the 15M-Indignados protests, the transformation of the party system with the emergence of new significant parties, major corruption scandals, the independence bid in Catalonia, or the popularization and mobilization of one of the world’s most prominent feminist movements.

For the first four waves, the study was the result of a collaboration agreement between the Spanish Centre for Sociological Research (Centro de Investigaciones Sociológicas CIS) and the Universitat Autònoma de Barcelona (UAB) Democracy, Elections and Citizenship research group (DEC). Established in 1963, the CIS is a Spanish public research institute that conducts scientific studies of Spanish society, primarily through periodic surveys. The DEC research group has been analyzing citizens’ political attitudes and behaviors through surveys since 2008. From waves 5 through 12, the study was carried out independently by the DEC research group.

## Methods

The dataset contains 20,991 observations, obtained from online surveys completed by 4,633 Spanish individuals over 12 waves distributed over a period of 10 years between 2010 and 2020. Since the 12^th^ wave, the panel has continued on a yearly basis, and it’s expected to run for the foreseeable future. Data from the 13th wave onward will be part of future releases. The project received ethical clearance from the Commission on Ethics in Animal and Human Experimentation (CEEAH) of the Universitat Autònoma de Barcelona (reference number CEEAH 5995).

### Survey description

The length of the online self-administered questionnaires varied across waves, with the median completion time ranging from 14 to 35 minutes. The dataset contains a total of 661 variables. The main topics covered by the questionnaire are included in Table 1 but more detailed information, including the questionnaires used in each wave, can be found in the public data repository. While most variables reflect respondents’ original answers to the survey questions, a few were created ex-post by recoding respondents’ answers or from other information (e.g., variables that record correct answers given to political knowledge questions). Note that while most questions are repeated across waves, not all variables are present in all 12 waves. The dataset does not include any direct identifiers of the respondents.

**Table 1:** Mains subjects included in the questionnaires (included in at least three waves).

Subjects	Indicators
Partisanship and voting behavior	Vote recall, probability to vote for main parties, closeness to a party, partisanship strength, trust in main parties, placement of main parties on different dimensions (left/right, Spanish nationalism, freedom vs order, taxes vs services).
Political engagement	Interest in politics, internal political efficacy, political participation (petitions, boycotts, contacts, donations, demonstrations), online political participation (donations, contacts, emails, petitions), political knowledge, emotions and feelings towards politics, sense of duty to vote, sense of duty to demonstrate, likelihood to vote, turnout, union membership.
Attitudes and predispositions	Left-right, order vs freedom, Spanish nationalism, identification with the state/region, populist attitudes, modern sexism, risk aversion, postmaterialist values.
Political trust	Social trust, in the president (prime minister), in the leader of the opposition, in the parliament, in the government, in banks, in the judiciary, in politicians, in unions, in the main parties, in the main leaders, in the European parliament, in the UE.
Issue positions	Most important problem (and associated emotions), European Union, climate change, abortion, gay adoption rights, prostitution, immigration (economic and cultural perceptions), territorial organization of the state, political correctness, order vs freedom, taxes vs. public services, need to reform the political system, perceptions of main parties’ positions on main issues.
Media consumption	Time exposed to TV every day, internet use, frequency of TV/radio/online/newspaper news consumption, social media (Twitter, Facebook, WhatsApp) use and trust.
Economic perceptions	Perceptions of country’s current economic situation, personal economic situation, future economic situation, emotions elicited by the economic situation, EU/Spanish government responsible for the economic situation, ability of government/opposition to deal with economic issues.
Great Recession	Opinions on austerity measures, emotions elicited by the economic crisis, shift in needs and habits, economic distress, poverty and social exclusion (EPICES).
Socio-demographics	Sex, age, size of municipality of residence, educational level, region, working situation, job type, partner’s job type, occupation, income, mortgage, public sector worker, living arrangements, household composition, number of children, childbirth.

### Survey sampling and administration

The panel includes twelve waves, fielded between 2010 and 2020. The fieldwork was carried out by the company Netquest, using CAWI (Computer Assisted Web Interviewing). The first wave was fielded in November 2010, followed by three waves at six-month intervals. Since 2012, the survey has been fielded annually around the month of June. The duration of the fieldwork ranged from eight days in wave six to 32 days in wave ten.

The panel is run over a non-probability sample of individuals drawn from the Netquest panel^7^. At the time the survey was first fielded (2010) internet use among older cohorts was limited. According to the Spanish National Statistics Institute (INE), in 2010, 36% of people aged 45 to 54, 62% of those between 55 and 64 years of age, and 84% of those between 65 and 75 in Spain had never used the internet, while percentages were under 14% for younger cohorts^8^. Hence the panel started by surveying only individuals with Spanish nationality and Spanish residence who were aged 16 to 44. Over time, the panel extended its age range to an upper limit of 58 in wave 12. From wave 5 onwards the lower limit was set at 18. Note that the panel is unbalanced, that is, not all individuals are observed for all waves.

Individuals provided consent for the use and public release of their survey responses by accepting the terms and conditions when registering on the Netquest platform. They were rewarded for their participation in the surveys with points (varying depending on the length of the questionnaire) that could be exchanged for material goods.

The selection of the sample in the first wave was made in proportion to the population parameters of the classification variables usually used in the sample designs of CIS surveys, applying quotas based on the crossing of sex and age (three categories: 16–24, 25–34 and 35–44 years old) and the crossing of municipality size (three categories: up to 50,000 inhabitants, 50,000 to 500,000 or more than 500,000) and regions (autonomous communities).

Refreshment samples were included in waves 2, 9, 10 and 12 to account for attrition and to improve sample representativeness. The fresh sample in the second wave was composed of individuals in the lower educational categories to compensate for the over-representation of individuals with a university education in the first wave. The quota structure of the other refreshment samples was based on sex and age, education, size of municipality and region - depending on the wave - and aimed to approximate the distribution of these variables in the Spanish population. (detailed information is provided in the methodological report included in the public data repository).

In waves 2, 3, and 4, only those who had participated in the previous wave were invited to complete the survey. From wave 5 onward, participants from any previous wave were recontacted, with the only exception being wave 9, for which invitations were sent only to those who had participated in at least one wave between the 5th and 8th waves. Table 2 shows the final composition of each wave.

**Table 2:** Fieldwork dates, size and composition of each wave.

	N	New sample (%)	From previous wave(s) (%)	Dates
Wave 1	2100	2100 (100)		17 Nov– 10 Dec 2010
Wave 2	2433	620 (25)	1813 (75)	11 – 25 May 2011
Wave 3	1979		1979 (100)	9 – 18 Nov 2011
Wave 4	1717		1717 (100)	11 – 30 May 2012
Wave 5	1757		1757 (100)	17 May – 4 June, 16 October 2013
Wave 6	1071		1071 (100)	5 – 12 May 2014
Wave 7	1015		1015 (100)	27 Apr – 8 May 2015
Wave 8	1040		1040 (100)	4 – 17 May 2016
Wave 9	1990	992 (50)	998 (50)	29 May – 15 June 2017
Wave 10	2128	504 (24)	1624 (76)	9 May – 9 Jun 2018
Wave 11	1748		1748 (100)	31 May – 11 Jun 2019
Wave 12	2013	417 (21)	1596 (79)	7 – 18 May 2020

Percentages are calculated based on the N of the corresponding wave.

As shown in Table 3, the likelihood of (temporarily) dropping out of the panel is associated with certain socio-demographic characteristics. Among the predictors considered, age and education exert the most consistent effects: younger and less educated respondents are more likely to drop out. In a few waves, women and panelists who spend less time online also exhibit lower retention rates. As with other individual-level panel data, the relationship between attrition and specific socio-demographic factors limits the generalizability of findings from longitudinal analyses. In the case of the POLAT Panel, however, these concerns are partially mitigated by two factors. First, respondents who drop out are allowed to rejoin the panel in later waves, making it possible to reintegrate previously lost panelists. Second, the most consistent predictors of attrition, such as age and education, are factors that can be more easily compensated for through the implementation of population representative quotas in refreshment samples. In contrast, more difficult-to-compensate factors like political interest do not appear to be related to attrition. The participation patterns shown in Table 4 indicate that more than 75% of participants took part in at least two waves of the panel, with more than 50% of respondents participating in at least four waves.

**Table 3:** Predictors of attrition (Logit models).

	All waves	Wave 2	Wave 3	Wave 4	Wave 5	Wave 6	Wave 7	Wave 8	Wave 9	Wave 10	Wave 11	Wave 12
Municipality size												
50.000–500.000	−0.05	−0.06	−0.10	0.04	0.06	−0.06	−0.17	0.11	−0.18	−0.02	−0.27*	0.09
	(0.04)	(0.15)	(0.12)	(0.15)	(0.14)	(0.12)	(0.18)	(0.16)	(0.17)	(0.12)	(0.11)	(0.13)
Over 500.000	0.08	0.00	0.04	0.22	0.12	0.05	0.14	0.13	−0.22	−0.11	−0.08	−0.06
	(0.05)	(0.18)	(0.15)	(0.18)	(0.18)	(0.12)	(0.15)	(0.21)	(0.22)	(0.13)	(0.13)	(0.16)
Female	0.14***	0.04	0.25*	0.08	0.12	0.25*	−0.05	0.11	−0.07	0.16	0.17	0.31*
	(0.04)	(0.13)	(0.11)	(0.14)	(0.12)	(0.10)	(0.14)	(0.15)	(0.16)	(0.10)	(0.10)	(0.12)
Age	−0.01**	−0.01	0.01	0.02+	−0.03*	−0.02**	−0.02+	−0.01	−0.01	−0.03***	−0.03***	−0.03***
	(0.00)	(0.01)	(0.01)	(0.01)	(0.01)	(0.01)	(0.01)	(0.01)	(0.01)	(0.01)	(0.01)	(0.01)
Education	−0.05***	−0.12**	−0.13***	−0.03	−0.10**	−0.03	0.01	0.01	−0.01	−0.06*	−0.08**	−0.00
	(0.01)	(0.04)	(0.03)	(0.04)	(0.03)	(0.03)	(0.04)	(0.04)	(0.04)	(0.03)	(0.03)	(0.03)
Employed	0.06	0.02	−0.04	−0.11	0.16	0.20+	0.14	0.04	0.18	0.06	0.14	−0.08
	(0.04)	(0.15)	(0.12)	(0.15)	(0.14)	(0.11)	(0.15)	(0.16)	(0.19)	(0.11)	(0.11)	(0.14)
Political interest	−0.01	−0.02	−0.02	−0.04	−0.10	−0.01	−0.15+	0.03	0.07	0.03	0.16**	−0.04
	(0.02)	(0.08)	(0.07)	(0.08)	(0.08)	(0.06)	(0.08)	(0.09)	(0.10)	(0.06)	(0.06)	(0.08)
Time on internet	−0.01	−0.06	−0.05	−0.03	−0.10*	−0.07+	0.07	−0.17**	0.01	0.05	0.07+	−0.02
	(0.01)	(0.05)	(0.04)	(0.05)	(0.05)	(0.04)	(0.05)	(0.05)	(0.06)	(0.04)	(0.04)	(0.05)
Constant	−0.78***	−0.74+	−1.11**	−2.28***	0.18	0.54	−0.12	−0.27	−1.05+	−0.06	−0.40	−0.16
	(0.13)	(0.42)	(0.37)	(0.48)	(0.43)	(0.35)	(0.49)	(0.53)	(0.58)	(0.33)	(0.32)	(0.40)
Observations	18887	2100	2417	1958	1717	1742	1047	1012	1028	1990	2128	1748
Pseudo *R*^2^	0.003	0.008	0.019	0.006	0.014	0.008	0.010	0.009	0.004	0.015	0.020	0.017

Standard errors in parentheses.

**Note:** DV: 0 = *No dropout*; 1 = *Dropout*. *Female* and *Employed* are dummy variables (0-1) that indicate the status of the respondent. The *Education* variable is a six-value scale (from *Primary education or less* to *University education or more*). The *Political interest* variable is a four-value scale that ranges from *Not at all interested* to *Very interested*. The *Time on the internet per day* variable is a six-value scale that ranges from *Less than 1* *hour* to *More than 8* *hours*. Reference category for *Municipality size*: < 50.000. + p < 0.1, *p < 0.05, **p < 0.01, ***p < 0.001.

**Table 4:** Patterns of (continuous or discontinuous) participation of the POLAT’s panelists.

	N	% of participants
One wave	1099	23.7
Two waves	516	11.1
Three waves	597	12.9
Four waves	732	15.8
Five waves	272	5.9
Six waves	223	4.8
Seven waves	178	3.8
Eight waves	213	4.6
Nine waves	189	4.1
Ten waves	185	4
Eleven waves	206	4.5
Twelve waves	223	4.8
Total sample	4633	100

As with any other panel survey data, in POLAT there is also the trade-off between observing within-individual variation over time and having a perfectly representative sample of the target population. The regular sample refreshments compensate for potential bias produced by attrition. Because the POLAT panel started over a decade and a half ago, at a time when a large share of older citizens did not use the Internet, the wave 1 sample is only representative of people born from 1965 onwards. Users of POLAT should be aware of these limitations.

## Data Records

The dataset and the related information material are stored as open access in the CORA repository (version 4.0)^9^. The dataset is in long format: each column represents a single variable, and stores respondents’ answers to a specific question across all waves. Each row captures all the answers provided by a respondent for a single wave. For waves 2, 9, 10 and 12, a dedicated variable distinguishes the members of the refreshment samples from recontacted respondents. The twelve waves of the survey were cleaned, harmonized and merged using STATA software.

The release includes different files. The dataset, including 661 variables and 20,991 observations, is provided in CSV format (“POLAT_dataset_1-12_v4.csv”) and in STATA 16 format (“POLAT_dataset_1-12_v4.dta”). These files are accessible using the following software: STATA 16, MS Excel, OpenOffice or any other spreadsheet software. Details on the fieldwork, refreshment samples, criteria for recontacting subjects, variables included in each wave, and changes from previous releases can be found in the methodological reports, available in English (“POLAT_MetReport_1-12_EN_v4.pdf”) and in Spanish (“POLAT_MetReport_1-12_ES_v4.pdf”). Section 5 of the Methodological Report outlines the changes implemented relative to the first release of the data, which included only the first six waves of the panel and the related materials in the original language (Spanish)^10^. The questionnaires are available in Spanish (“POLAT_questionnaires_1-12_ES_v4.pdf”) and English (“POLAT_questionnaires_1-12_EN_v4.pdf”), each supplemented by a corresponding codebook: “POLAT_codebook_1-12_ES_v4.html” in Spanish and “POLAT_codebook_1-12_EN_v4.pdf” in English.

## Technical Validation

The Netquest panel is built following the highest quality standards and has an ISO 20252 certification. Self-registration is not permitted: it is not possible for a random Internet user to register on the database without having been previously invited. According to the company, this avoids the professionalization of panelists and minimizes the effects of self-selection. Potential respondents receive a personal invitation on their profile to complete a survey. When the candidates complete this initial survey, they receive an individual and unique invitation to become a member of the Netquest panel, and then they are subsequently invited to participate in surveys such as the POLAT Panel.

As mentioned in the Methods section, regular refreshment samples were included to improve the representativeness of the sample and account for attrition. Additionally, each survey in the panel included one or more ‘trap’ questions designed to identify and screen out inattentive respondents. An analysis of the impact of these trap questions can be found here^11^.

To ensure high-quality question wording, a threefold strategy was employed. First, standard items—such as voting behavior, ideological orientations or issue positions—were drawn from established CIS studies and recognized international survey projects such as the Comparative Study of Electoral Systems (CSES) and the European Social Survey (ESS). Second, question batteries designed to measure specific concepts were based on those used in previous recognized research. These include batteries on topics such as grievances in times of crisis^12^, sexism^13^, populism^14^, and authoritarianism^15^. Third, we also included some innovative items to measure new concepts, such as political correctness^16^.

To assess the validity of the indicators, we compared the distribution of selected items from the first six waves of the POLAT Panel and from two concurrent face-to-face surveys conducted on representative samples of the Spanish population that used similar question wording as well as rigorous sampling techniques and tried and tested field protocols (the CIS monthly Barometer and the ESS)^17^. The comparison of the ideological self-placement question between the second wave of the POLAT Panel (conducted in May 2011) and the fifth round of the ESS (April-July 2011) revealed nearly identical average values and similar distributions. Likewise, respondents’ evaluations of the Spanish economy showed a very similar trend across the first six waves of the POLAT Panel and the corresponding CIS monthly barometers.

## Supplementary information

STATA code

## Data Availability

The twelve waves of the survey were prepared for merging using the STATA software. The STATA code used to produce Tables 2–4 is provided in the supplementary material.
